# *Máscarasdo Bem:* An analysis of face-mask performance from a volunteer mask-making initiative in Ribeirão Preto, Brazil

**DOI:** 10.1016/j.puhip.2021.100094

**Published:** 2021-02-08

**Authors:** Karina F.S. Leite, Kezi Cheng, Shailabh Kumar, Emilia M.P.C. Chayamiti, Márcia Costa, Maryann C. Tung, Karen M.L. Morejón, Cátia H.D. Salomão, Stella C. Lopes, Henrique Pacini

**Affiliations:** a University of São Paulo, São Paulo, Brazil; b Harvard University, Cambridge, MA, USA; c Secretaria da Saúde, Municipality of Ribeirão Preto, Brazil; d Stanford University, Stanford, CA, USA; e UNIMED Ribeirão Preto, Brazil

**Keywords:** Face masks, COVID-19, Community response, Brazil

## Abstract

**Objectives:**

This study examines the response of a group of volunteers in Ribeirão Preto, Brazil, as the city faced an unprecedented demand for face masks during the onset of the COVID-19 crisis in 2020. The performance of artisanal-produced masks was compared with industry equivalents.

**Study design:**

Case report with comparative testing.

**Methods:**

A comparison was made between two parallel projects that produced single-use masks for healthcare workers and reusable masks for the community. Mask samples were tested for filtration efficiency (FE) and breathability (pressure drop).

**Results:**

Results for FE averaged 40–60% for healthcare masks and 10% for community masks; both types of masks were tested for particle sizes of 0.3 ​μm.

**Conclusions:**

While performance was inferior to standard comparators, the masks investigated in this study afforded a level of protection in the absence of alternatives, especially in non-aerosol generating contexts. The findings of this study are useful for communities with limited resources in other developing countries. In addition, insights can be gained from the experiences in Ribeirão Preto in terms of how to respond to future health emergencies.

## 1 Introduction

The coronavirus disease 2019 (COVID-19) pandemic, as a result of severe acute respiratory syndrome coronavirus 2 (SARS-CoV-2) infection, was initially detected in Wuhan, China, in December 2019 and subsequently spread to Brazil in March 2020 [1]. Municipalities in Brazil had to respond to the public health emergency in a context of limited resources and mounting logistical and procurement challenges. In Brazil, the public health system is part of the *Sistema Único de Saúde* (SUS) national network, which is provides universal and free health care [2,3]. In Ribeirão Preto (population 704,000), a city in the state of São Paulo, the public health network is made up of 52 health clinics, varying in size and complexity [4]. This network is run by 3100 healthcare workers, who, due to COVID-19, suddenly required a large additional volume of personal protection equipment (PPE), including face masks, to reduce infection risks [5,6].

Face masks are essential items of PPE and can be classified into the following three main types: (1) N95 masks confer the most protection from viral spread for both the wearer and others, but are also the most expensive; (2) surgical masks confer partial protection for the wearer and others; and (3) cloth masks offer limited protection to the wearer, primarily protecting others from viral spread, but are much more affordable [7].

A serious municipal budgetary crisis and worldwide shortages of PPE compounded the difficulties facing health authorities in Ribeirão Preto [8,9]. In response, nurses linked to the health secretariat of Ribeirão Preto organised a network of volunteers and mask-making production commenced in April 2020. Government guidelines were followed for the recommended materials and designs for making face masks [10,11]. Two initiatives were launched: the first, producing single-use masks for healthcare workers to act as an emergency substitute for surgical masks; and the second, creating reusable community/cloth masks for distribution to SUS users [12,13].

This study documents the response in Ribeirão Preto, Brazil, to the lack of PPE during the early stages of the COVID-19 pandemic; these results are in the context of a developing country, where affordability and functionality play an important role. Independent test results are presented on samples of the masks produced, estimating their filtration efficiency and breathability (pressure drop).

### 1.1 Masks for healthcare workers

In the public health system of Ribeirão Preto, the normal demand (i.e. burn rate) for surgical masks was approximately 300 units/month prior to the pandemic. After the onset of COVID-19 in March 2020, this rose to 3000 units/month. The municipal stocks of surgical face masks, which were scheduled to last for 6 months, unexpectedly ran out within a few days. Previous studies have shown that health-related public procurement in Brazil is notoriously slow, limiting rapid government responses to pandemic-induced PPE shortages [14]. In this context, and given the scarcity of N95 or surgical masks, a group of volunteers produced masks for use in the municipal public health system.

**Fig. 1:**
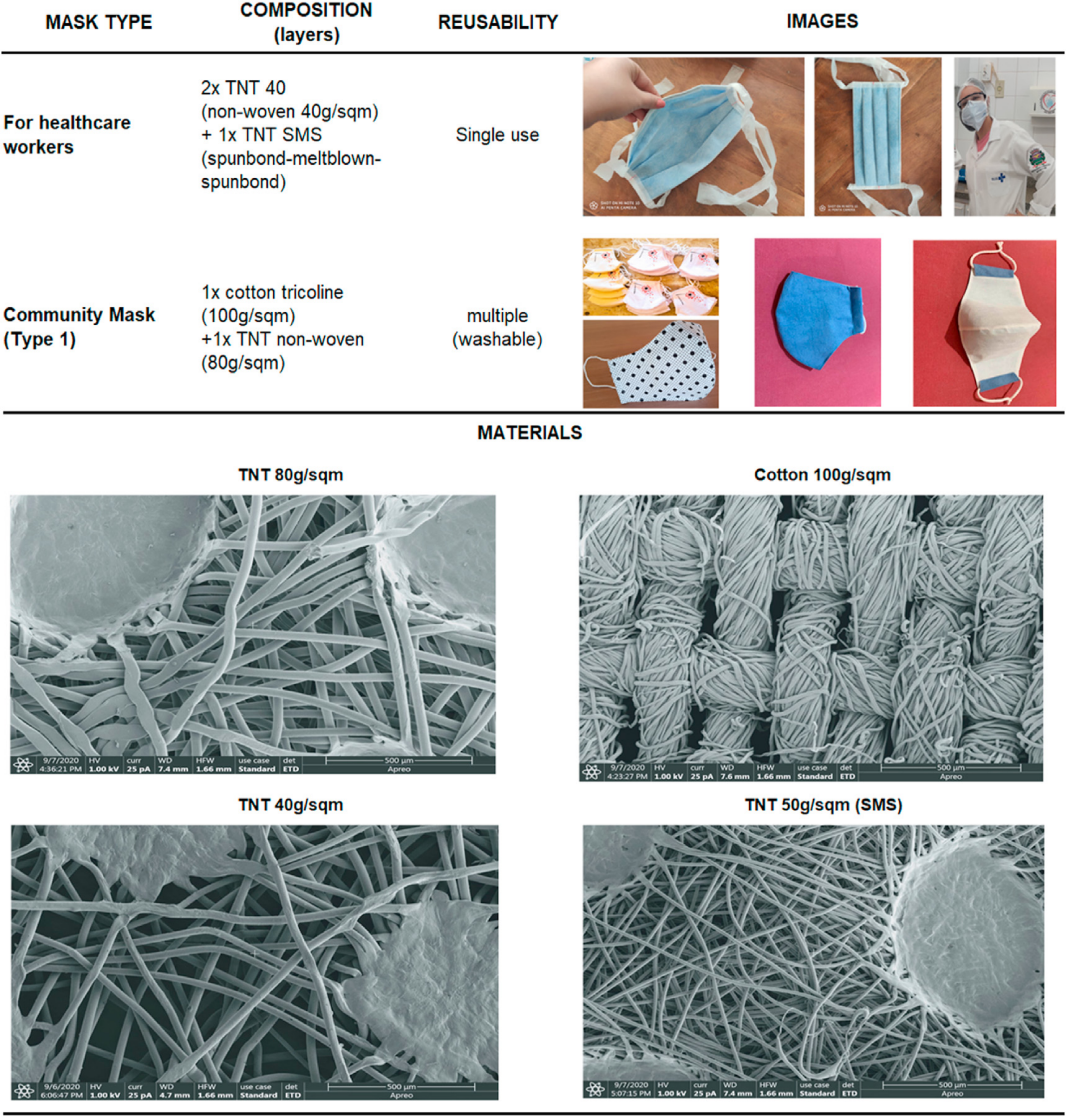
Masks and materials tested. Scanning electronic microscope imaging performed at a horizontal field width of 1.66 ​mm. TNT, *tecido-não-tecido.*

The organisers used social networks and work channels to recruit 50 volunteers and used workspace available at the, then-idle, industrial sewing facilities at the *Servico Nacional de Aprendizagem Comercial* (SENAC), a national vocational school with units in Ribeirão Preto [15]. Masks were sewn in a controlled environment, following protocols for workplace sanitising and personal hygiene. Work was carried out 5 days a week, in two shifts, following official guidelines [10,11,16]. Complementing reference standards, volunteers received constant guidance from infection control professionals on workplace safety and on the design of surgical masks. The materials used to create the masks for healthcare workers were (see Fig. 1):

● Inner and outer layer: TNT (*tecido-não-tecido*/non-woven fabric, spunbond) 100% polypropylene, 40 ​g/sqm.
● Middle layer: TNT SMS (*tecido-não-tecido* spunbond/non-woven fabric - meltblown - spunbond) 100% propylene, 50 ​g/sqm.
● Coated aluminium wire (for the nasal clip).
● Standard sewing material (thread and industrial machines borrowed by SENAC).

The inner and outer layers of TNT 40 were cut to 34 ​× ​21 ​cm and the SMS middle-layer filter was 21 ​× ​17 ​cm. In making the mask body, TNT 40 was folded in half in its largest dimension and the SMS filter was placed between the two layers of the folded TNT. A seam to join the three layers was made in an overlock machine at the top of the mask. The coated aluminium wire was embedded in a fold of about 1 ​cm ​at the top, which was subsequently sewn on a straight machine. Three pleats were made, from top to bottom, so that the height of the mask was between 8 and 9 ​cm. Two side seams were made to maintain the pleats. Then, an 80 ​cm strip of TNT 40 ​g was sewn on each side (for upper and lower lashing). The mask had three layers in total.

The three-month project helped supply the PPE requirements of the city’s public health units and some other public institutions, such as nursing homes, blood banks, public hospitals, the local police force and the Mobile Emergency Care Support team (SAMU) ambulances. While the specific roles of mask performance and usage frequency factors in reducing COVID-19 community transmission are still being actively researched, mask usage is now widely recommended [17]. This initiative helped to safeguard local healthcare workers until accelerated public procurement and the improvement of market conditions allowed stocks of conventional PPEs to be replenished. In total, 58,551 face masks for healthcare workers were produced.

### 1.2 Masks for the community (general population)

The majority of users of the public health system in Brazil are economically or socially vulnerable [18]. While substantial labour reallocation from garment industries to mask-making business occurred in 2020, many users of SUS are still struggling to afford general-use face masks [19]. In April 2020, the city of Ribeirão Preto made the use of face masks compulsory in public spaces, which prompted demand for low-cost and reusable face mask options [20]. In response to this situation, a second project was launched, named *Máscaras* *do Bem for Ribeirão Preto - Comunidade*, focusing on the production of community masks for low-income and/or vulnerable users of SUS.

In order to identify volunteers and raise funds for the purchase of materials, groups were set up on Facebook and Whatsapp. Approximately 170 individuals responded to the volunteer requests, some helping in mask-making activities, others split among support roles, such as transportation logistics, fundraising, donors and process administration. While traditionally nurse-led initiatives primarily involve females, this volunteer group included 21 men who collaborated in the construction of moulds, the cutting and distribution of masks, and other tasks.

Masks were distributed, at no-cost, by health service managers. Beneficiaries were patients, family members and caregivers. Similar to the initiative for healthcare worker mask production, the community masks were also based on recommended designs and materials [10,11]. The community face masks had two layers and the materials used were (see Fig. 1):

● Outer layer: 100% cotton *tricoline* 100 ​g/sqm.
● Inner layer: TNT (*tecido-não-tecido*/non-woven fabric, spunbond) 100% propylene, 80 ​g/sqm.

Unlike the masks for healthcare workers, the delivery of community masks to the population posed challenges. For optimal impact, the reusable masks needed to quickly reach families and people with the greatest social vulnerability. This problem was, once again, tackled through social media, by the establishment of a support network. The volunteer group facilitated both upstream (bringing materials to seamstresses) and downstream (taking masks to beneficiaries by car, bicycle or interpersonal channels). In April 2020, the group produced 9906 masks, in May 9616 and in June a further 948 masks were produced. In total 20,524 reusable community masks were produced, all of which were distributed to those in need. The initiative ended in June 2020, with production results far exceeding initial goals.

## 2 Methods

Initiatives similar to *Máscaras* *do Bem* in Ribeirão Preto have been documented in many other Brazilian cities, such as Araçatuba, Porto Feliz, Curitiba, Xaxim and Colina [[21], [22], [23], [24], [25]]. While all initiatives were based on designs and materials recommended by authorities, a common feature among the initiatives is the absence of independent testing. Few centres with testing capabilities are available to the public in Brazil, with the exception of the *Respire!* consortia at the University of São Paulo [26]. Given the large uptake of mask production (of similar design) within Brazil, mask performance data are essential. In this study, independent performance data, according to parameters accepted in the literature, for the healthcare and community masks are presented.

Respiratory infections occur through the transmission of virus-containing droplets (>5–10 ​μm) and aerosols (≤5 ​μm) exhaled from infected individuals during breathing, speaking, coughing and sneezing [27]. Previous studies have estimated the COVID-19 virus diameter to be in the range of 50–150 ​nm [28]. Masks produced by the *Máscaras* *do Bem* initiative were tested at the Prakash lab at Stanford University, based on sealed samples sent from Ribeirão Preto, Brazil. The tests used a Fluke optical particle counter with readings for 0.3 ​μm, 0.5 ​μm, 1.0 ​μm, 2.5 ​μm and 5 ​μm. Using laboratory room air, there were only sufficient particles for tests with particles measuring 0.3 ​μm, 0.5 ​μm and 1.0 ​μm. Tests with larger-sized particles were not performed due to insufficient particles. Tests for filtration efficiency (FE) were made at 10 ​cm/s face velocity. Pressure drop was tested at various velocities; results reported in this study were at ~6.6 ​cm/s face velocity.

FE tests performed on the *Máscaras* *do Bem* masks at the University of São Paulo (USP) under its *Respire!* initiative were different to FE tests performed by the US National Institute for Occupational Safety and Health (NIOSH). Instead of sodium chloride (NaCl) aerosols, which are the NIOSH standard, tests performed in this analysis used ambient air as the source for particles of diverse sizes. This test allows an estimation of the FE of the masks [29].

Masks were tested for FE with particles of 0.3 ​μm, which is the standard used by NIOSH tests and is considered the particle size that is most likely to pass through filter materials [30,31]. Smaller and larger particles get trapped more easily because of dominating diffusion and impaction [32]. Particles larger than 1.0 ​μm tend to be affected significantly by gravity, while particles under 0.1 ​μm are most affected by Brownian motion, which causes adherence of small air particles to the fibres in the filter material[33].

## 3 Results

**Fig. 2:**
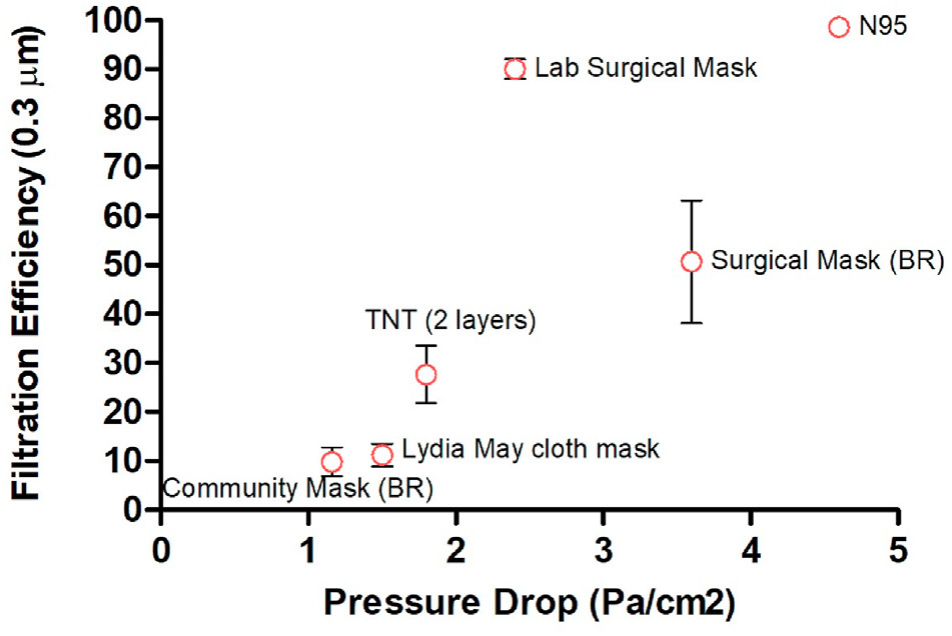
Test results for filtration efficiency and pressure drop. BR, Brazil; TNT, *tecido-não-tecido.*

Test results indicated that masks produced for healthcare workers, labelled Surgical Mask (BR) in Fig. 2, had filtration efficiencies ranging from 40% to 60%. This was lower than industry-produced ASTM-certified masks which were used as comparators. ASTM level 2 masks had filtration performances close to 90% (Fig. 2, label Lab Surgical Mask). Pressure drop readings also indicated superior breathability performance for the ASTM comparator.

FE results for the community masks from Ribeirão Preto were around 10%, which is equivalent to the comparator (Lydia May cloth mask, Fig. 2). This is expected, since Lydia May masks are made very similarly, having one layer of cotton and the second layer of non-woven shopping bag material (e.g. TNT). Previous studies indicate that even low FE-performing masks can be useful for infection control, especially when coupled with handwashing and social distancing [34].

The second measurement made was pressure drop, which measures breathability. Higher pressure drop values indicate more resistance to airflow and increased difficulty breathing [35]. Pressure drop results indicate that the community masks had the best breathability performance, which could translate into more regular usage.

## 4 Discussion

This study describes a community response to produce face masks for healthcare workers and the community during the onset of the COVID-19 pandemic in Brazil. Amid a context of market scarcity for industry-made equivalents, volunteer-led efforts, organised by local nurses, produced significant numbers of face masks to compensate for the lack of conventional PPE supplies. Rapid organisational results were achieved through the establishment of social network groups and through the mobilisation of assets, such as production sites, machinery, and specialised labour, which and who were rendered idle due to pandemic-related restrictions on vocational schools and stores. In total, 79,021 masks were produced based on official standards.

N95 masks and high-quality surgical masks are undisputedly the best face masks for health care settings. However, when such masks are not available, masks with observed FE ranging from 40 to 60% at 0.3 ​μm and better at larger particle sizes can provide a degree of protection, especially in settings where healthcare workers are not involved in aerosol-generating procedures.

Test results show that the masks produced for healthcare workers had performances inferior to the industry standards, but in an acceptable range for FE and pressure drop with selected comparators. Results were broadly in line with what was expected, given test parameters and materials available. Breathability results suggest a slightly better performance for community masks, indicating a level of comfort in their use. The results from this study will be useful for ongoing efforts to improve community-based responses to future health emergencies, as well as for the further development of official guidelines on artisanal PPE production.

## Ethical approval

Not required.

## Funding

This work received financial support from UNIMED Ribeirão Preto. Part of this work was performed at the Stanford Nano Shared Facilities (SNSF), supported by the National Science Foundation under award ECCS-1542152. No other funding sources were involved.

## Declaration of competing interest

All authors declare they have no conflict of interest in the development of this article.

## References

[1] WHO (2020). WHO timeline - COVID-19. https://www.who.int/news-room/detail/27-04-2020-who-timeline---covid-19.

[2] Planalto (1990) Lei 8080 de 19 de Setembro de 1990, establishing the Sistema Único de Saúde. http://www.planalto.gov.br/ccivil_03/leis/l8080.htm.

[3] Jairnilson Silva Paim (2018). Sistema Único de Saúde (SUS) aos 30 anos. Ciência & saúde coletiva. https://www.scielo.br/scielo.php?pid=S1413-81232018000601723&amp;script=sci_arttext.

[4] Ribeirão Preto (2020). Organigramm of public health clinics (Unidades Básicas de Saúde) of Ribeirão preto. https://www.ribeiraopreto.sp.gov.br/files/ssaude/pdf/organograma-ubs.pdf.

[5] CDC (2019). Cloth face mask guidance. https://www.cdc.gov/coronavirus/2019-ncov/prevent-getting-sick/cloth-face-cover-guidance.html.

[6] CDC (2019). Face masks for COVID-19 protection. https://www.cdc.gov/coronavirus/2019-ncov/hcp/ppe-strategy/face-masks.html.

[7] N95Decon (2020). Face masks: understanding the difference. https://static1.squarespace.com/static/5e8126f89327941b9453eeef/t/5f31c960e483de6a393939f0/1597098339651/200803_Mask_Comparison_v5.pdf.

[8] Estado (2017). Crise faz prefeito congelar pagamentos em Ribeirão Preto. https://politica.estadao.com.br/noticias/geral,crise-faz-prefeito-congelar-pagamentos-em-ribeirao-preto,10000097541.

[9] Livingston Edward, Desai Angel, Berkwits Michael (2020). Sourcing personal protective equipment during the COVID-19 pandemic. https://jamanetwork.com/journals/jama/article-abstract/2764031.

[10] ABNT (2020). Máscaras de proteção respiratória de uso não profissional. Guia de requisitos básicos para métodos de ensaio, fabricação e uso. https://www.abntcatalogo.com.br/norma.aspx?ID=442968.

[11] ANVISA (2020). General instruction for assembly of non-professional face masks. Agência Nacional de Vigilância Sanitária. http://portal.anvisa.gov.br/documents/219201/4340788/NT+M%C3%A1scaras.pdf/bf430184-8550-42cb-a975-1d5e1c5a10f7.

[12] G1 (2020). Volunteers form group to make masks to be donated to hospitals in Ribeirão Preto. https://g1.globo.com/sp/ribeirao-preto-franca/noticia/2020/03/27/voluntarios-se-unem-para-fazer-mascaras-que-serao-doadas-a-hospitais-de-ribeirao-preto-sp.ghtml.

[13] Revide (2020). Volunteers have produced over 38.000 masks for domestic use for communities in Ribeirão Preto. https://www.revide.com.br/noticias/coronavirus/voluntarios-ja-produziram-38-mil-mascaras-de-uso-domestico-para-comunidades-de-rp/.

[14] Bevilacqua Gabriela, Farias Rocha, Blatt Mareni, Raquel Carine (2011). https://www.scielosp.org/article/rsp/2011.v45n3/583-589/.

[15] SENAC (2020). Fashion and swing courses for begginers. Serviço Nacional de Aprendizagem Comercial. Ribeirão Preto unit. https://www.sp.senac.br/cursos-livres/curso-de-modelagem-e-costura-para-iniciantes.

[16] Ministry of Health (2020). RDC nº 356, de 23 de março de 2020 - requisitos para a fabricação, importação e aquisição de dispositivos médicos identificados como prioritários para uso em serviços de saúde, em virtude da emergência de saúde pública internacional relacionada ao SARS-CoV-2. http://portal.anvisa.gov.br/documents/10181/5809525/RDC_356_2020_.pdf/0655c7ae-8c47-4be9-bf0d-4c7b8df03e4e.

[17] Stutt Richard O.J. H., Retkute Renata, Bradley Michael, Gilligan Christopher A., Colvin John (2020). A modelling framework to assess the likely effectiveness of facemasks in combination with “lock-down” in managing the COVID-19 pandemic. Proceedings of the Royal Society A.

[18] Boing Alexandra Crispim, Bertoldi Andréa Dâmaso, Boing Antonio Fernando, Bastos João Luiz, Peres Karen Glazer (2013). Access to medicines in the public sector: analysis of users of the Brazilian unified national health system. Cad. Saúde Pública.

[19] IPEA (2020). Extensão Universitária, Economia Solidária e Geração de Oportunidades no contexto da COVID-19: Uma visão a partir de três experiências concretas no território brasileiro. http://repositorio.ipea.gov.br/handle/11058/10189.

[20] Acidadeon (2020). Usage of facemasks becomes mandatory on 22 April 2020. https://www.acidadeon.com/ribeiraopreto/cotidiano/mundo/NOT,0,0,1512122,uso+de+mascara+e+obrigatorio+a+partir+desta+quarta+22.aspx.

[21] Hojemais (2020). https://www.hojemais.com.br/aracatuba/noticia/cotidiano/municipio-mobiliza-voluntarios-para-fabricacao-de-mascaras-para-toda-a-populacao.

[22] G1 (2020). Volunteers produce cloth masks to distribute to inhabitants during pandemic in Porto Feliz. https://g1.globo.com/sp/sorocaba-jundiai/noticia/2020/04/07/voluntarios-produzem-mascaras-de-tecido-para-distribuir-aos-moradores-durante-pandemia-em-porto-feliz.ghtml.

[23] Príncipe Pequeno (2020). Volunteers produce masks and uniforms for Pequeno Principe hospital in Curitiba. http://pequenoprincipe.org.br/noticia/voluntarios-confeccionam-mascaras-e-uniformes-para-profissionais-do-pequeno-principe/.

[24] Xaxim (2020). With the support of volunteers, city produces masks to protect against COVID-19. https://www.xaxim.sc.gov.br/noticias/index/ver/codMapaItem/13800/codNoticia/613079.

[25] ODiario (2020). Volunteers at the social solidarity of Colina fund make facemasks. https://www.odiarioonline.com.br/noticia/92680/fundo-social-de-solidariedade-de-colina-confecciona-mascaras.

[26] InovaUSP (2020). Projeto Respire - interdisciplinary research for innovative solutions. https://inova.usp.br/respire/.

[27] Prather Kimberly, Wang Chia, Schooley Robert T. (2020). Reducing transmission of SARS-CoV-2. Science.

[28] Bar-On Yinon, Flamholz Avi, Phillips Rob, Milon Ron (2020). SARS-CoV-2 (COVID-19) by the numbers. https://elifesciences.org/articles/57309.

[29] Cramer Avilash, Tian Enze, Galanek Mitchell (2020). Assessment of the qualitative fit test and quantitative single-pass filtration efficiency of disposable N95 masks following gamma irradiation. JAMA Network Open.

[30] IEST (2016). https://www.iest.org/Standards-RPs/Recommended-Practices/IEST-RP-CC001.

[31] Dietz Leslie, Horve Patrick, Coil David, Fretz Mark, Eisen Jonathan, Van den Wymelenberg Kevin (2020). 2019 novel coronavirus (COVID-19) pandemic: built environment considerations to reduce transmission. https://www.ncbi.nlm.nih.gov/pmc/articles/PMC7141890/#B51.

[32] N95Decon (2020). Technical document for public use of medical masks and cloth masks. https://static1.squarespace.com/static/5e8126f89327941b9453eeef/t/5ea3b5859bc8f31a11f3deb5/1587787141808/2020-04-24_N95DECON_Face_Mask_Technical_Report_v1_final.pdf.

[33] Jayaweera Mahesh, Perera Hasini, Gunawardana Buddhika, Manatunge Jagath (2020). Transmission of COVID-19 virus by droplets and aerosols: a critical review on the unresolved dichotomy. Environ. Res..

[34] Sharma Suresh K., Mishra Mayank, Mudgal Shiv K. (2020). Efficacy of cloth face mask in prevention of novel coronavirus infection transmission: a systematic review and meta-analysis. J. Educ. Health Promot..

[35] Kim Jung-Hyun, Roberge Raymond J., Powell Jeffrey B., Shaffer Ronald E., Ylitalo Caroline M., Sebastian John M. (2015). Pressure drop of filtering facepiece respirators: how low should we go?. Int. J. Occup. Med. Environ. Health.

